# A Critical Role of CDKN3 in Bcr-Abl-Mediated Tumorigenesis

**DOI:** 10.1371/journal.pone.0111611

**Published:** 2014-10-31

**Authors:** Qinghuang Chen, Ke Chen, Guijie Guo, Fang Li, Chao Chen, Song Wang, Grzegorz Nalepa, Shile Huang, Ji-Long Chen

**Affiliations:** 1 College of Animal Sciences, Fujian Agriculture and Forestry University, Fuzhou, Fujian, China; 2 CAS Key Laboratory of Pathogenic Microbiology and Immunology, Institute of Microbiology, Chinese Academy of Sciences (CAS), Beijing, China; 3 Department of Pediatrics and Division of Pediatric Hematology-Oncology, Herman B Wells Center for Pediatric Research, Indiana University School of Medicine, Indianapolis, Indiana, United States of America; 4 Department of Medical and Molecular Genetics, Indiana University School of Medicine, Indianapolis, Indiana, United States of America; 5 Department of Biochemistry and Molecular Biology, Louisiana State University Health Sciences Center, Shreveport, Louisiana, United States of America; University of Pecs Medical School, Hungary

## Abstract

CDKN3 (cyclin-dependent kinase inhibitor 3), a dual specificity protein phosphatase, dephosphorylates cyclin-dependent kinases (CDKs) and thus functions as a key negative regulator of cell cycle progression. Deregulation or mutations of CDNK3 have been implicated in various cancers. However, the role of CDKN3 in Bcr-Abl-mediated chronic myelogenous leukemia (CML) remains unknown. Here we found that CDKN3 acts as a tumor suppressor in Bcr-Abl-mediated leukemogenesis. Overexpression of CDKN3 sensitized the K562 leukemic cells to imanitib-induced apoptosis and dramatically inhibited K562 xenografted tumor growth in nude mouse model. Ectopic expression of CDKN3 significantly reduced the efficiency of Bcr-Abl-mediated transformation of FDCP1 cells to growth factor independence. In contrast, depletion of CDKN3 expression conferred resistance to imatinib-induced apoptosis in the leukemic cells and accelerated the growth of xenograph leukemia in mice. In addition, we found that CDKN3 mutant (CDKN3-C140S) devoid of the phosphatase activity failed to affect the K562 leukemic cell survival and xenografted tumor growth, suggesting that the phosphatase of CDKN3 was required for its tumor suppressor function. Furthermore, we observed that overexpression of CDKN3 reduced the leukemic cell survival by dephosphorylating CDK2, thereby inhibiting CDK2-dependent XIAP expression. Moreover, overexpression of CDKN3 delayed G1/S transition in K562 leukemic cells. Our results highlight the importance of CDKN3 in Bcr-Abl-mediated leukemogenesis, and provide new insights into diagnostics and therapeutics of the leukemia.

## Introduction

Chronic myelogenous leukemia (CML) is a hematopoietic malignancy characterized by the presence of the Philadelphia chromosome that arises from a reciprocal translocation between the *Bcr* gene on chromosome 22 and the *c-Abl* gene on chromosome 9, resulting in the formation of *Bcr-Abl* oncogene [1], [2]. Previous studies have revealed that deregulation of multiple signaling pathways associated with cell survival and proliferation, including phosphoinositide-3-kinase (PI3K)/AKT, RAS, and Janus kinase (JAK)/signal transducer and activator of transcription (STAT), underlies Bcr-Abl-induced tumorigenesis [3]–[5]. However, the precise mechanisms by which Bcr-Abl causes leukemogenesis are not fully clarified.

Dysregulation of cell cycle causes aberrant cell proliferation, which potentiates genomic instability and cancer development [6]–[8]. It is well known that Bcr-Abl expression in hematopoietic cells promotes cell cycle progression from G1 to S phase, leading to cytokine-independent proliferation [9], [10]. Bcr-Abl may downregulate expression of cyclin-dependent kinase (CDK) inhibitor p27^Kip1^ not only by suppressing its mRNA expression but also by enhancing its protein degradation through the PI3K/AKT-mediated proteasome pathway, resulting in activation of CDKs to accelerate cell cycle progression [11]–[13]. Although alterations in cell cycle progression and cell proliferation have been implicated in Bcr-Abl-mediated tumorigenesis, the precise contribution of relevant signaling molecules to the development of CML remains to be further defined [9].

As a member of the dual specificity protein phosphatase family, CDKN3 (CDK inhibitor 3, also called CDI1 or KAP) plays a key role in regulating cell division [8], [14]–[17]. The gene encoding CDKN3 protein is located on chromosome 14q22 [18]. It is well known that CDKN3 can specifically dephosphorylate and inactivate CDK2, thereby inhibiting G1/S cell cycle progression [19]. CDKN3 also interacts with CDK1 (also known as Cdc2 in fission yeast) and controls progression through mitosis by dephosphorylating CDC2 at Thr161 and consequently reducing phosphorylation of CKβ at Ser209 [17]. CDKN3 has been suggested to function as a tumor suppressor, and its loss of function was found in a variety of cancers [17], [20]. For example, downregulation of CDKN3 has been found in glioblastoma [17]. Loss of CDKN3 has also been observed in hepatocellular carcinoma [20]. Contradictorily, CDKN3 is highly expressed in breast and prostate cancers, and blocking CDKN3 expression can inhibit the transformation [21]. In addition, elevated levels of CDKN3 occur in renal cell carcinoma (RCC), and enforced CDKN3 expression significantly enhances cell proliferation and xenograft tumor growth in renal cancer cells, suggesting an oncogenic function of CDKN3 [22]. While more work is needed to dissect the role of the CDKN3 in cancer, these findings suggest that CDKN3 may potentially function either as an oncogene or a tumor suppressor. Interestingly, several spliced transcript variants encoding different isoforms of CDKN3 were found in diverse cancers, implying that these isoforms may be associated with specific tumor formation [23], [24]. Despite the importance of CDKN3 in tumorigenesis, how CDKN3 plays a role in Bcr-Abl-induced leukemia and the mechanism by which CDKN3 functions to impact Bcr-Abl-mediated cellular transformation are largely unknown.

Here we found that CDKN3 acted as a tumor suppressor in Bcr-Abl-induced tumorigenesis. Overexpression of CDKN3 delayed G1/S transition, sensitized imatinib-induced apoptosis in K562 leukemic cells, and inhibited the growth of xenografted leukemias in nude mice. In addition, we observed that forced expression of CDKN3 significantly impaired the efficiency of Bcr-Abl-mediated FDCP1 cellular transformation. Furthermore, we revealed that CDKN3 reduced the cell survival by disrupting CDK2-dependent expression of XIAP. Together, our experiments establish an important role for CDKN3 in Bcr-Abl-mediated leukemogenesis, and provide a potential new therapeutic target for treatment of Abl-positive malignancies.

## Materials and Methods

### Cell lines and cell culture

Cell lines 293T and K562 were purchased from American Type Culture Collection (ATCC) and grown in Dulbecco's modified Eagle medium (DMEM) or RPMI1640 supplemented with 10% fetal bovine serum (FBS) and antibiotics (penicillin and streptomycin) as previously described [4]. SUP-B15 cell line was obtained from Cell Resource Center, Chinese Academy of Sciences in Shanghai and cultured in IMEM supplemented with 20% FBS and antibiotics. FDCP1 cell line was purchased from ATCC and grown in RPMI1640 supplemented with 10% fetal bovine serum containing antibiotics and 3 ng/ml murine IL3. CDKN3-overexpressing K562 cells were generated by infecting the cells with retroviruses encoding FLAG-tagged CDKN3 using the pMSCV-IRES-GFP vector as previously described [5]. Short hairpin RNA (shRNA)-expressing K562 or SUP-B15 cells were generated by infection of the cells with lentiviruses expressing specific shRNA in pSIH-H1-GFP vector as described previously [5].

### Antibodies and reagents

The following antibodies were used in this study: anti-FLAG (Sigma, Saint Louis, MO, USA); anti-CDK2 and anti-phospho-CDK2 Thr160 (Santa Cruz Biotechnology, Dallas, TX, USA); anti-c-Abl (Merck Millipore, Billerica, MA, USA); anti-XIAP (Cell Signaling, Danvers, MA, USA). All other antibodies were obtained as described previously [25]. Thymidine and nocodazole were purchased from Sigma, RNase Inhibitor was obtained from Thermo Scientific (Waltham, MA, USA), and murine IL3 was purchased from PEPRO TECH (Rocky Hill, NJ, USA).

### Construction of CDKN3 expressing and specific shRNA expressing vectors

FLAG-tagged CDKN3 was subcloned into pMSCV-IRES-GFP to generate pMSCV-CDKN3-IRES-GFP. CDKN3 mutant (CDKN3-C140S) devoid of the phosphatase activity and CDK2 dominant-negative mutant (CDK2-D145N) were generated using a QuickChange site-directed mutagenesis kit (Stratagene, La Jolla, CA, USA). The shRNA-expressing constructs were generated by subcloning shRNA oligonucleotides into BamH I and EcoR I sites of pSIH-H1-GFP vector (System Biosciences, Mountain View, CA, USA) as previously described [26]. The shRNA sequence targeting CDKN3 is 5′-GCCGCCCAGTTCAATACAAAC-3′.

### Reverse transcription PCR (RT-PCR) and real-time PCR

Total RNA was extracted using TRizol reagent (Invitrogen, Carlsbad, CA, USA). cDNA was synthesized using GoScript™ Reverse Transcription System (Promega, Madison, WI, USA) and oligo (dT) primers (Takara, Dalian, China) by following the manufacturer's instruction. Briefly, 4 µg RNA was mixed with 0.5 µg oligo (dT) primers and nuclease-free water to a final volume of 5 µl. This mixture was heated at 70°C for 5 minutes and then immediately chilled on ice for 5 minutes, followed by addition of 15 µl GoScript™ reverse transcription mix (1.5 mM MgCl_2_, 0.5 mM each dNTP, 20 units of RiboLock RNase Inhibitor, 160 units of GoScript Reverse Transcriptase). The reaction was then performed by annealing at 25°C for 5 minutes, extending at 42°C for 60 minutes and inactivating reverse transcriptase at 70°C for 15 minutes. The synthesized cDNA was amplified by PCR using rTaq DNA polymerase (Takara, Tokyo, Japan) with specific primers following the manufacturer's instruction. The quantitative real-time PCR was conducted with SuperReal PreMix Plus kit (TIANGEN, Beijing, China) following the manufacturer's instruction. The following primers were used for both PCR and real-time PCR: human CDKN3 forward, (5′-GGACTCCTGACATAGCCAGC-3′) and reverse (5′-CTGTATTGCCCCGGATCCTC-3′); human XIAP forward, (5′-TGAAAATAGTGCCACGCAGTCT-3′) and reverse (5′-CTGGCCAGTTCTGAAAGGACTT-3′). Expression level of β-actin or GAPDH was used as a control.

### Western blotting

Western blotting was conducted as previously described [27], [28]. Briefly, cells were treated as indicated in the figure legends, harvested, and lysed for protein collection. Samples were then separated on SDS-polyacrylamide gel, transferred to a nitrocellulose membrane, and probed with antibodies as indicated.

### Apoptosis and cell viability assay

Cell apoptosis assay was performed using KeyGEN Annexin V-APC/propidium iodide (PI) Apoptosis Detection kit (KeyGEN BioTECH, Nanjing, China) according to the manufacturer's instructions. Briefly, cells were treated with 5 µM or 10 µM imatinib for the indicated times. The samples were washed with ice cold phosphate-buffered saline (PBS) and stained with 2.5 µg/ml Annexin V-APC and 1 µg/ml propidium iodide. The samples were then examined by fluorescence-activated cell sorter (BD Bioscience, San Jose, CA, USA) as previously described [5], [29]. For cell viability assay, cells were treated with 10 µM imatinib for indicated time, washed with ice cold PBS, stained with 1 µg/ml of propidium iodide, and examined by fluorescence-activated cell sorter as described previously [5], [29]. All the data were analyzed by FCS Express V3 Software (De Novo Software, Thornhill, Ontario, Canada).

### Bcr-Abl-mediated FDCP1 transformation

FDCP1 cells were infected with retroviruses carrying *Bcr-Abl* oncogene by spin infection with 8 µg polybrene at 32°C for 2 hours. The infected cells were suspended in RPMI1640 containing 10% FBS and seeded in 96-well plates (each plate was seeded with 4×10^6^ cells equally). The transformation efficiency was scored by counting the number of wells that displayed cytokine-independent growth 2 weeks after infection as described previously [27].

### Cell cycle synchronization and cell cycle analysis

K562 cells were synchronized at the G1/S transition as previously described [30], [31]. In brief, cells were treated with 2 mM of thymidine for 13.5 h and then released for 9 h, followed by treatment with thymidine for 13.5 h. Cells were released for 2.5 h and subjected to flow cytometry analysis. To obtain cells in mitosis, cells were treated with thymidine for 13.5 h and then released for 6 h, followed by treatment with 100 ng/ml of nocodazole for 6 h. Cells were released for 2 or 4 h, and subjected to flow cytometry analysis. Cell cycle analysis was performed as previously described [30], [31]. Briefly, cells were collected at the indicated time, fixed in 75% ethanol at −20°C overnight, then washed and incubated with propidium iodide (5 mg/ml with 0.1% RNase A) for 30 min. The samples were analyzed with a fluorescence-activated cell sorter (BD Bioscience).

### Nude mouse xenograft experiments

Female nude mice (5–6 weeks old) were obtained from Vital River Laboratories (VRL) (Beijing, China). Nude-mouse injection was performed as previously described [4]. Tumor growth was monitored and measured in volume (length×height×width) at the indicated time points. Bioluminescent imaging was performed to detect tumors originating from the GFP-expressing cells. Mice were anesthetized using 2% isoflurane and imaged using a cooled CCD camera. Images were quantified as photons/s using the indigo software (Berthold Technologies, Bad Wildbad, Germany).

### Ethics statement

The mouse experimental design and protocols used in this study were approved by “the regulation of the Institute of Microbiology, Chinese Academy of Sciences of Research Ethics Committee” (Permit Number: PZIMCAS2013008). All mouse experimental procedures were performed in accordance with the Regulations for the Administration of Affairs Concerning Experimental Animals approved by the State Council of People's Republic of China.

### Statistical analysis

Results were expressed as mean values ± standard error (mean ± SE). Statistical significance was determined by Student's t-test. A level of *P*<0.05 was considered to be significant.

## Results

### CDKN3 negatively regulates K562 leukemic cell survival in the presence of imatinib

**Figure 1:**
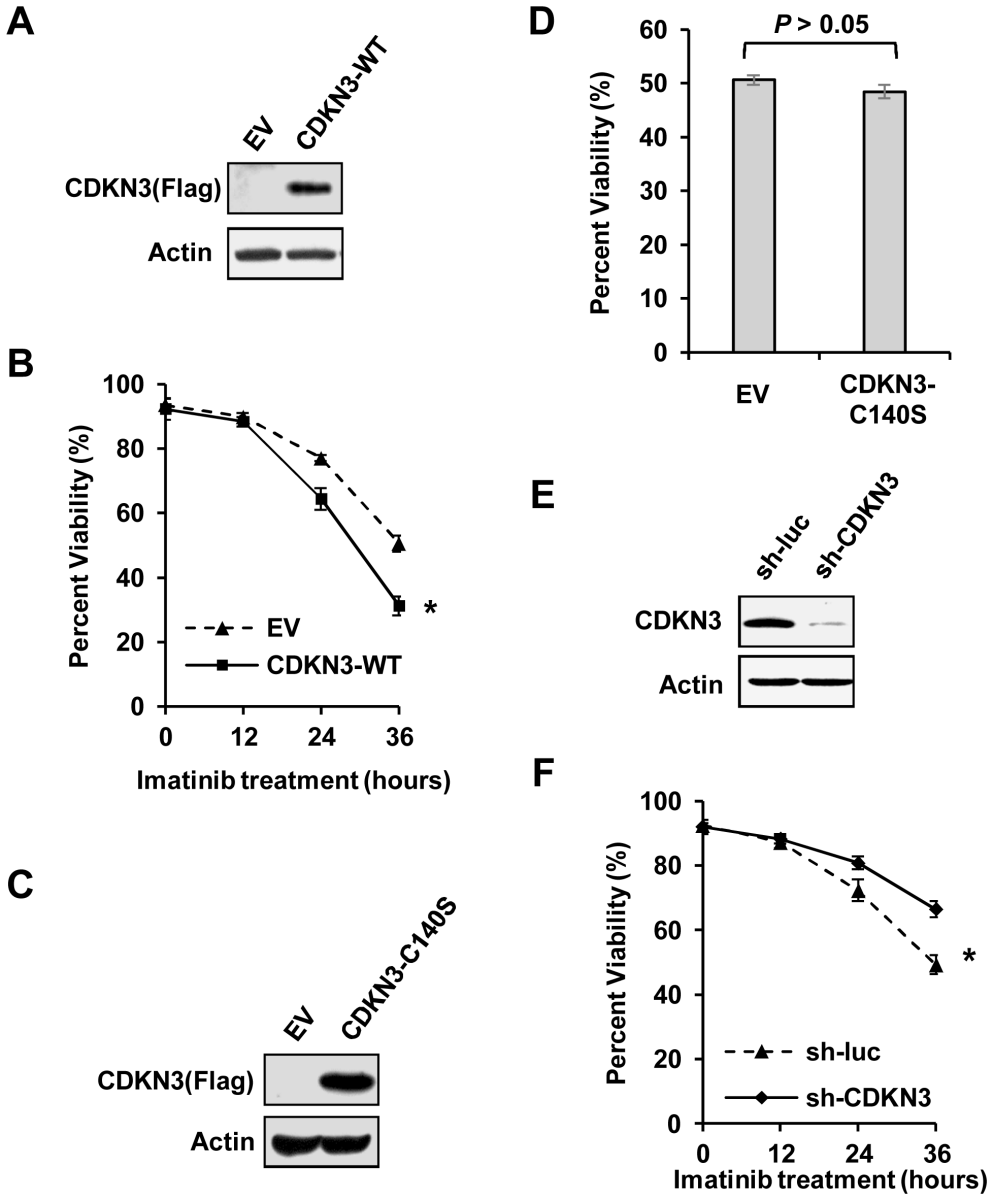
CDKN3 negatively regulates K562 cell survival. (A) Expression of CDKN3 in K562 cells stably overexpressing FLAG-tagged wild type CDKN3 (CDKN3-WT) or empty vector (EV) was detected by Western blotting using indicated antibodies. (B) K562 cells stably overexpressing CDKN3-WT or EV were treated with 10 µM of imatinib for the indicated time. Samples were stained with Annexin V-APC and PI, examined by flow cytometry and analyzed by FCS Express V3. Plotted are results from three independent experiments. Error bars represent SEM, *n* = 3; *P<0.05. (C) Shown is an immunoblot examining FLAG-tagged CDKN3-C140S in K562 cells ectopically expressing CDKN3 mutant (CDKN3-C140S) or empty vector (EV). (D) Cell viability of K562 cells expressing CDKN3-C140S or EV was assessed by flow cytometry after treatment with 10 µM of imatinib for 36 h. Samples were stained with Annexin V-APC and PI. Plotted are results from three independent experiments. Error bars represent SEM, *n* = 3. (E) Shown is an immunoblot examining shRNA-based knockdown of CDKN3. (F) K562 cells stably expressing sh-luc or sh-CDKN3 were treated with 10 µM imatinib for the indicated time. Samples were then stained with Annexin V-APC and PI, followed by flow cytometry analysis. Plotted are results from three independent experiments. Error bars represent SEM, *n* = 3; *P<0.05.

Deregulation or mutation of CDKN3 has been associated with a variety of human cancers [17], [20], [22], [23], [32], but it is unknown whether it plays a role in Bcr-Abl-induced tumorigenesis. To address this issue, we generated K562 leukemic cells stably expressing wild type CDKN3 (CDKN3-WT), or empty vector control (EV) (Figure 1A, Figure S1). These cells were treated with 10 µM of imatinib for up to 36 h, followed by Annexin V and PI staining and flow cytometry analysis. We found that under these culture conditions, approximately 50% of the control cells remained viable after incubation with imatinib for 36 h. In contrast, only approximately 31% of CDKN3-WT overexpressing cells were viable under the same imatinib treatment (Figure 1B), although overexpression of CDKN3 had no significant effect on the cell survival in the absence of imatinib (Figure S2A). To rule out the possibility of off-target responses caused by the imatinib dose at 10 µM, we also treated K562 cells with 5 µM imatinib for 36 h. Similarly, overexpression of CDKN3 significantly reduced the cell viability under this condition as compared with the control (Figure S2B). Importantly, there was no significant difference in cell survival between the control cells and cells expressing CDKN3 mutant (CDKN3-C140S) devoid of the phosphatase activity, in response to imatinib treatment (Figures 1C and 1D). These data suggest that CDKN3 overexpression promotes imatinib-induced apoptosis in K562 cells, and that the phosphatase activity of CDKN3 is required for its function in regulating leukemic cell survival in the presence of imatinib.

To further confirm the role of CDKN3 in regulating the survival of Bcr-Abl-transformed leukemic cells, K562 cell lines stably expressing shRNA targeting CDKN3 (sh-CDKN3) or luciferase (sh-luc) control were generated. Examination by Western blotting, RT-PCR and real time PCR showed that expression of CDKN3 was strongly diminished in cells expressing related shRNA (Figure 1E, and Figure S1). However, depletion of CDKN3 had no significant effect on the apoptosis of K562 cells without imatinib treatment (Figure S2C). These cells were then treated with 10 µM imatinib for indicated times and analyzed for cell survival. As expected, our results showed that approximately 49% of the control cells remained viable after treatment with imatinib for 36 h, while approximately 66% of the CDKN3 knockdown cells were viable under the same condition (Figure 1F), indicating a critical role for CDKN3 in imatinib-induced apoptosis of K562 leukemic cells. In addition, we also treated these K562 cells with 5 µM imatinib for 36 h. CDKN3 knockdown resulted in an increase in cell viability after imatinib treatment as compared to the control (Figure S2D). To better clarify the function of CDKN3 in Bcr-Abl tumorigenesis, we employed another Bcr-Abl positive cell line SUP-B15 that expresses comparable level of CDKN3 to K562 (Figure S3A). SUP-B15 cells stably expressing shRNA against CDKN3 or luciferase were generated (Figure S3B). The cells were then treated with 5 µM imatinib for 24 h and stained with Annexin V and PI. Similarly, depletion of CDKN3 significantly increased the cell viability after treatment with imatinib (Figure S3C). Taken together, these data implicate CDKN3 as a negative regulator of leukemic cell survival in the presence of imatinib.

### Altering CDKN3 expression has profound effects on Bcr-Abl-dependent tumor growth in a nude mouse xenograft model

**Figure 2:**
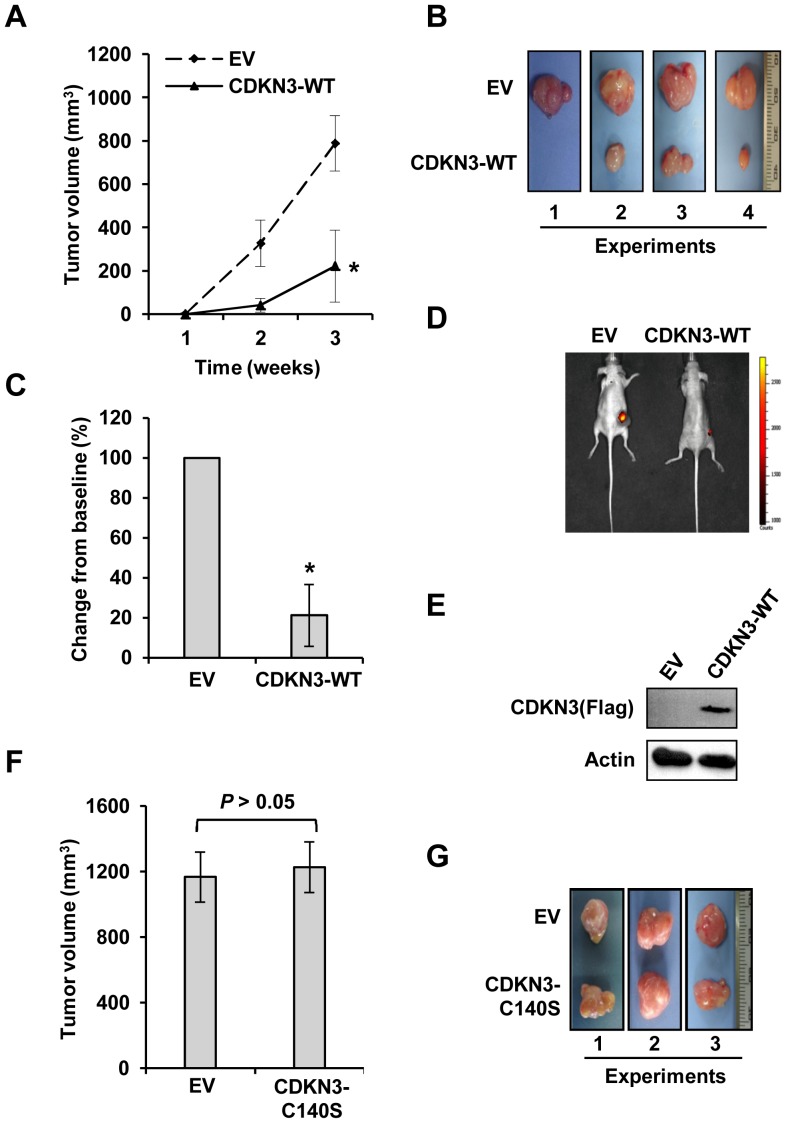
Overexpression of CDKN3 dramatically inhibits K562 xenografted tumor growth in nude mice. (A) Nude mice were subcutaneously injected with K562 cells stably expressing CDKN3-WT or EV. The tumor volumes were measured at indicated time points. Plotted are results from three independent experiments. Error bars, SEM; *n* = 9; *P<0.05. (B) Tumors were excised from mice. Shown are representative images from four independent experiments with similar results. (C) Relative volume of tumors excised from nude mice injected with K562 cells expressing CDKN3-WT or EV (control). The average volume of control tumors is set to 100%. Error bars, SEM; *n* = 9; *P<0.05. (D) Over a 21-day period after inoculation, tumors formed by control or CDKN3-WT overexpressing K562 cells were measured by bioluminescent imaging. Shown are representative images from at least three independent experiments with similar results. (E) CDKN3 expression in representative tumors expressing CDKN3-WT or EV was examined by Western blotting. (F) Nude mice were subcutaneously injected with K562 cells stably expressing CDKN3-C140S or EV. The tumor volumes were measured at indicated time points. Shown are volumes of tumors excised from nude mice injected with K562 cells expressing CDKN3-C140S or EV. Plotted are results from three independent experiments. Error bars, SEM; *n* = 9. (G) Tumors from nude mice injected with K562 cells expressing CDKN3-C140S or EV were excised from mice. Shown are representative images from three independent experiments with similar results.

To understand whether CDKN3 regulates Bcr-Abl-mediated tumorigenesis *in vivo*, nude mice were subcutaneously injected with K562 cells stably expressing CDKN3-WT or empty vector as control. Tumor volumes were measured each week after inoculation. Remarkably, we observed that the tumors formed by K562 cells overexpressing the CDKN3 phosphatase grew clearly slower than those formed by control cells (Figure 2A and 2B). Statistical analysis revealed that the tumor growth was significantly inhibited by exogenous expression of CDKN3-WT in K562 cells (Figures 2C). This finding was validated via bioluminescent imaging analysis (Figure 2D) and confirmed in three independent experiments. Additionally, Western blotting analysis demonstrated the overexpression of CDKN3 in the slow growth tumors (Figure 2E). In contrast, no significant difference was observed in the growth of tumors formed by K562 cells overexpressing phosphatase-dead CDKN3-C140S and control cells (Figures 2F and 2G). Together, these findings revealed that ectopic expression of wild type CDKN3, but not the phosphatase-deficient CDKN3-C140S mutant, significantly impeded the growth of the Bcr-Abl-driven K562 cells in the nude mouse xenograft model.

**Figure 3:**
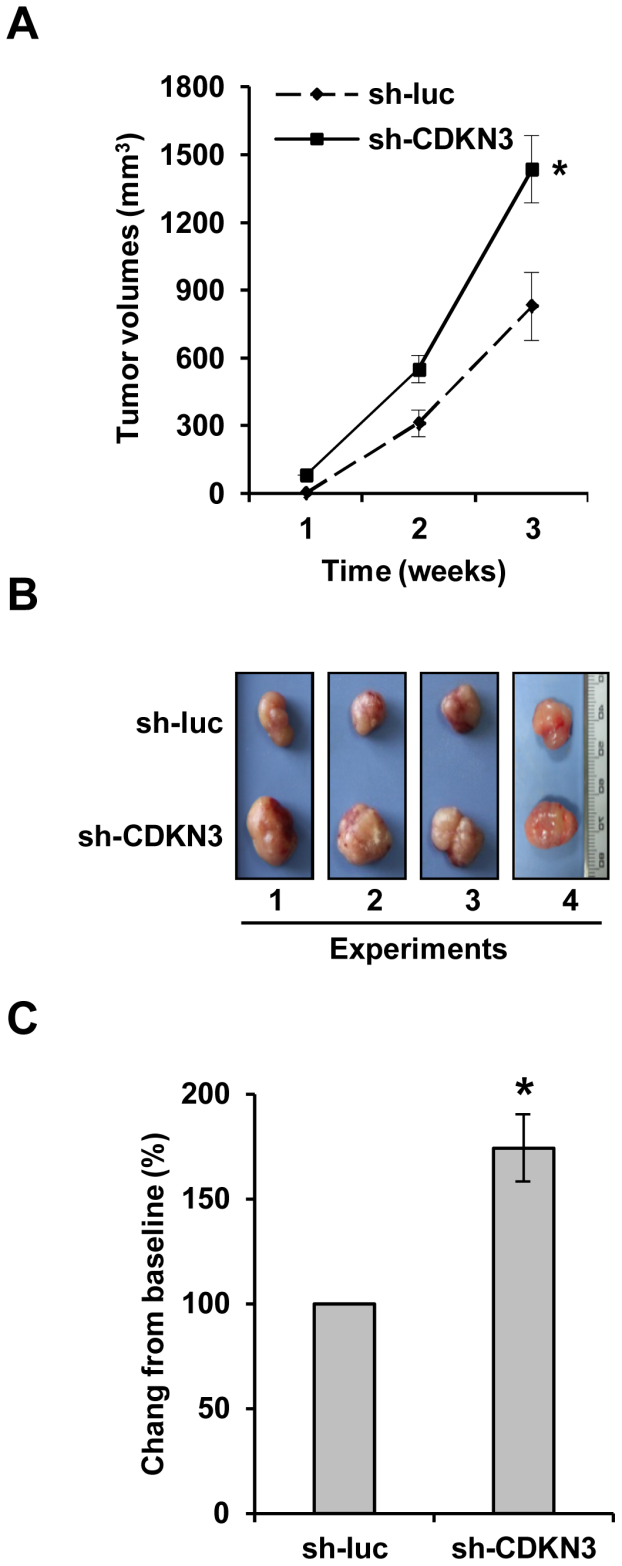
CDKN3 deficiency promotes K562 xenografted tumor growth in nude mice. (A) Nude mice were subcutaneously injected with K562 cells stably expressing sh-CDKN3 or sh-luc (control). The tumor volumes were measured at indicated time points. Plotted are results from three independent experiments. Error bars, SEM; *n* = 9; *P<0.05. (B) Tumors were excised from mice. Shown are representative images from four independent experiments with similar results. (C) Relative volume of tumors excised from nude mice injected with K562 cells expressing sh-luc (control) or sh-CDKN3. The average volume of control tumors is set to 100%. Error bars, SEM; *n* = 9; *P<0.05.

Since overexpression of CDKN3 inhibited the growth of leukemia *in vivo*, we hypothesized that decreased CDKN3 expression may promote the growth of leukemic tumors. To address this possibility, nude mice were injected subcutaneously with K562 cells stably expressing shRNA against CDKN3 (sh-CDKN3) or luciferase (sh-luc) control. As hypothesized, we found that silencing CDKN3 expression greatly promoted K562 xenografted tumor growth in nude mice (Figure 3A and 3B). Statistical analysis showed that a significant enhancement of tumor growth was induced by the knockdown of CDKN3 in K562 cells (Figures 3C). These results further indicate that CDKN3 phosphatase acts as a tumor suppressor in Bcr-Abl-mediated tumorigenesis.

### Forced expression of CDKN3 significantly reduced the efficiency of Bcr-Abl-mediated transformation of FDCP1 cells to growth factor independence

**Figure 4:**
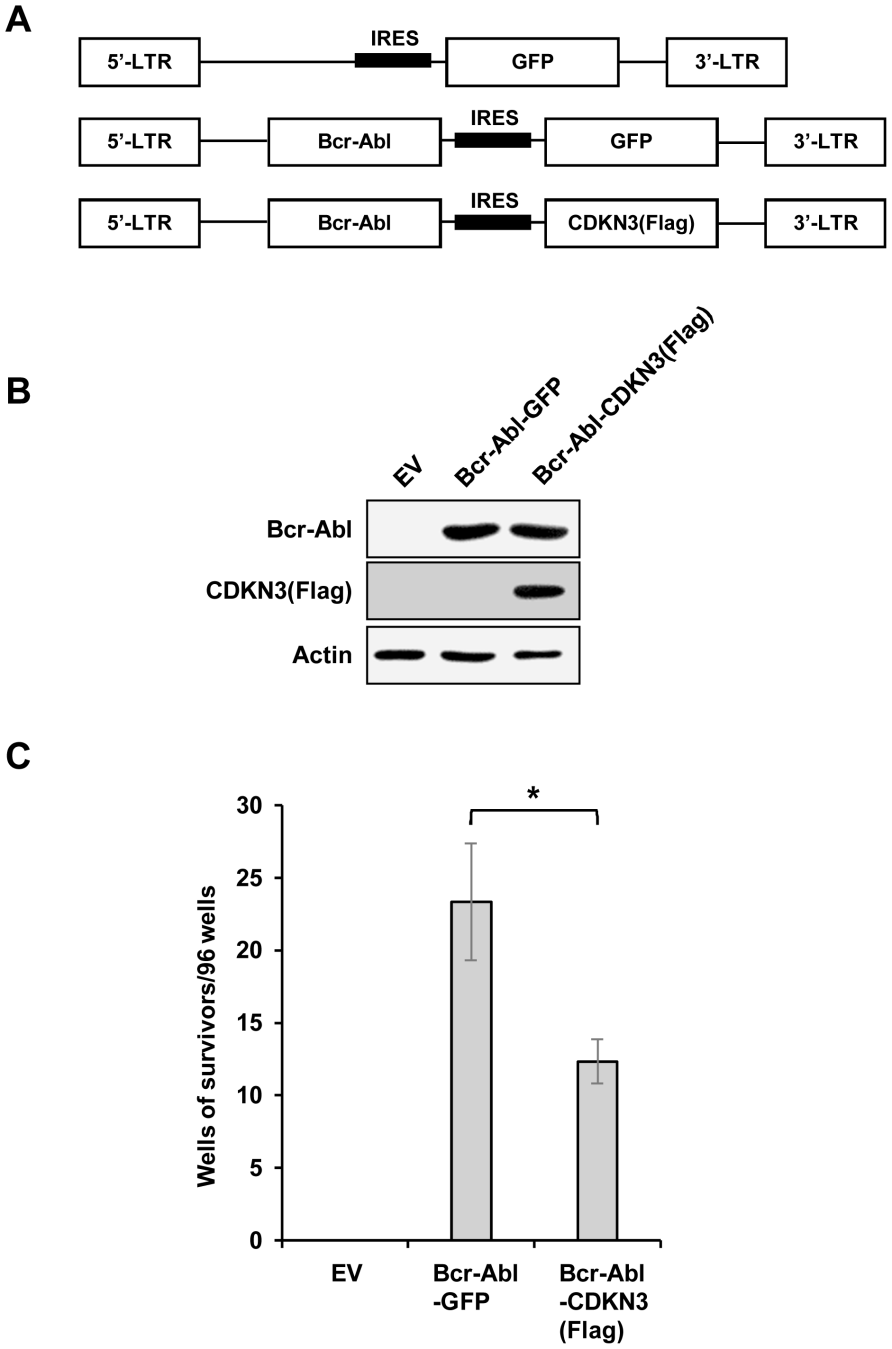
Overexpression of CDKN3 significantly reduces the efficiency of Bcr-Abl-mediated FDCP1 cell transformation. (A) Shown are constructs of bicistronic retroviruses carrying Bcr-Abl and GFP, Bcr-Abl and CDKN3-Flag, or empty vector (EV). (B) 293T cells were transfected with the plasmids described in (A). Cells were harvested after 36 hours and protein expression was detected by Western blotting using indicated antibodies. (C) FDCP1 cells were infected with retroviruses carrying empty vector, Bcr-Abl and GFP, or Bcr-Abl and CDKN3-Flag, and plated on 96-well plates. Transformation efficiency was assessed as described in Materials and Methods. Plotted are the results from three independent experiments. Error bars, SEM; *n* = 3; *P<0.05.

To better define the role of CDKN3 in Bcr-Abl-mediated cellular transformation, we generated bicistronic retroviruses encoding Bcr-Abl and either GFP or CDKN3 (Figure 4A and 4B). Equal titer of these viruses was used to infect FDCP1 cells. Efficiency of the viruses to transform FDCP1 cells to growth factor independence was then assessed by counting the number of wells containing Bcr-Abl-transformed cell clones. FDCP1 cells infected with viruses carrying Bcr-Abl-IRES-GFP showed an average result of 23 wells/96-well plate which displayed IL-3-independent growth of cell clones. Interestingly, the exogenous expression of CDKN3 remarkably impaired the transformation efficiency of Bcr-Abl to 12 wells/96-well plate (Figure 4C). These data provided strong evidence that CDKN3 significantly inhibited Bcr-Abl-induced cellular transformation and might function as a key suppressor in Bcr-Abl-mediated tumorigenesis.

### CDKN3 negatively regulates leukemia cells survival by disrupting CDK2-dependent XIAP expression

**Figure 5:**
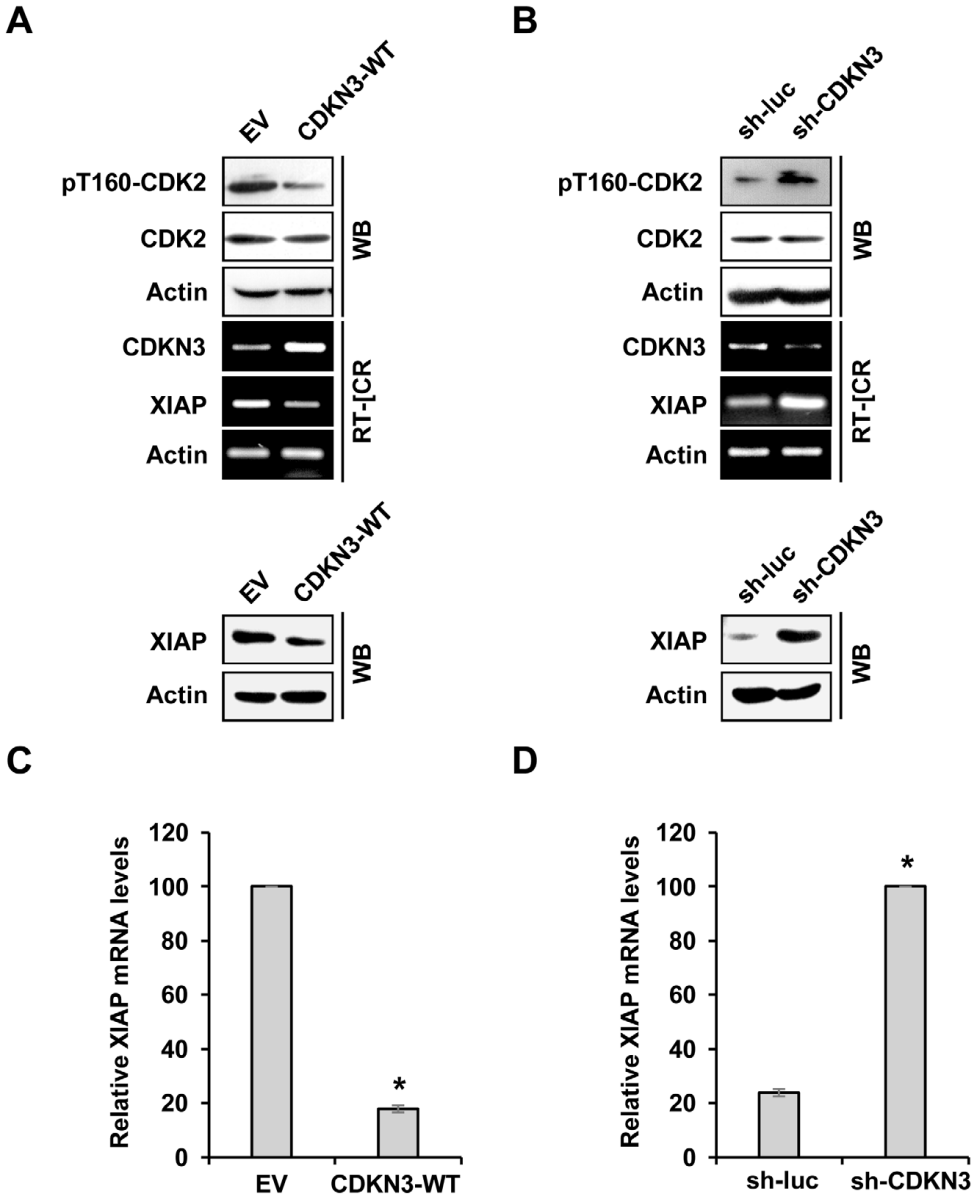
CDKN3 negatively regulates phosphorylation of CDK2 and expression of XIAP. (A) The levels of the indicated genes and proteins were detected by RT-PCR and Western blotting, respectively, in K562 cells overexpressing CDKN3-WT or EV. (B) Experiments were performed as described in (A). Shown are data from K562 cells expressing shRNA targeting CDKN3 or luciferase. (C) The mRNA expression of XIAP was detected by real-time PCR in K562 cells expressing CDKN3-WT or EV. Plotted are the results from three independent experiments. Error bars, SEM; *n* = 3; *P<0.05. (D) Experiments were performed as described in (C). Plotted are the results from three independent experiments using K562 cells expressing shRNA targeting CDKN3 or luciferase. Error bars, SEM; *n* = 3; *P<0.05.

Our experiments presented above demonstrated that the phosphatase activity of CDKN3 is essential for its pro-apoptotic activity in the CML leukemia cells. Previous studies have revealed that CDKN3 could dephosphorylate cyclin-dependent kinase 2 (CDK2) and thus modulate cell proliferation [23], [33]. It has been shown that CDK2-dependent expression of anti-apoptotic protein XIAP may account for the inefficient apoptosis in tumor cells including Bcr-Abl expressing cells [34]–[36]. Thus, we hypothesized that CDKN3 may inhibit the Bcr-Abl-mediated tumorigenesis at least partially by dephosphorylating and inactivating CDK2 to inhibit CDK2-dependent XIAP expression. To test this hypothesis, we examined the activation of CDK2 and the expression of XIAP in K562 cells expressing CDKN3-WT, CDKN3 specific shRNA, empty vector control, or luciferase specific shRNA control. The results showed that forced expression of CDKN3-WT reduced the levels of CDK2 phosphorylation at Thr160. Importantly, we observed that overexpression of CDKN3-WT caused a marked decrease in mRNA and protein expression of XIAP (Figure 5A and 5C). Conversely, depletion of CDKN3 increased CDK2 phosphorylation at Thr160 and the expression of XIAP (Figure 5B and 5D). These data suggest that CDKN3 might modulate leukemic cell survival at least partially through regulating CDK2-dependent expression of the anti-apoptotic regulator, XIAP.

**Figure 6:**
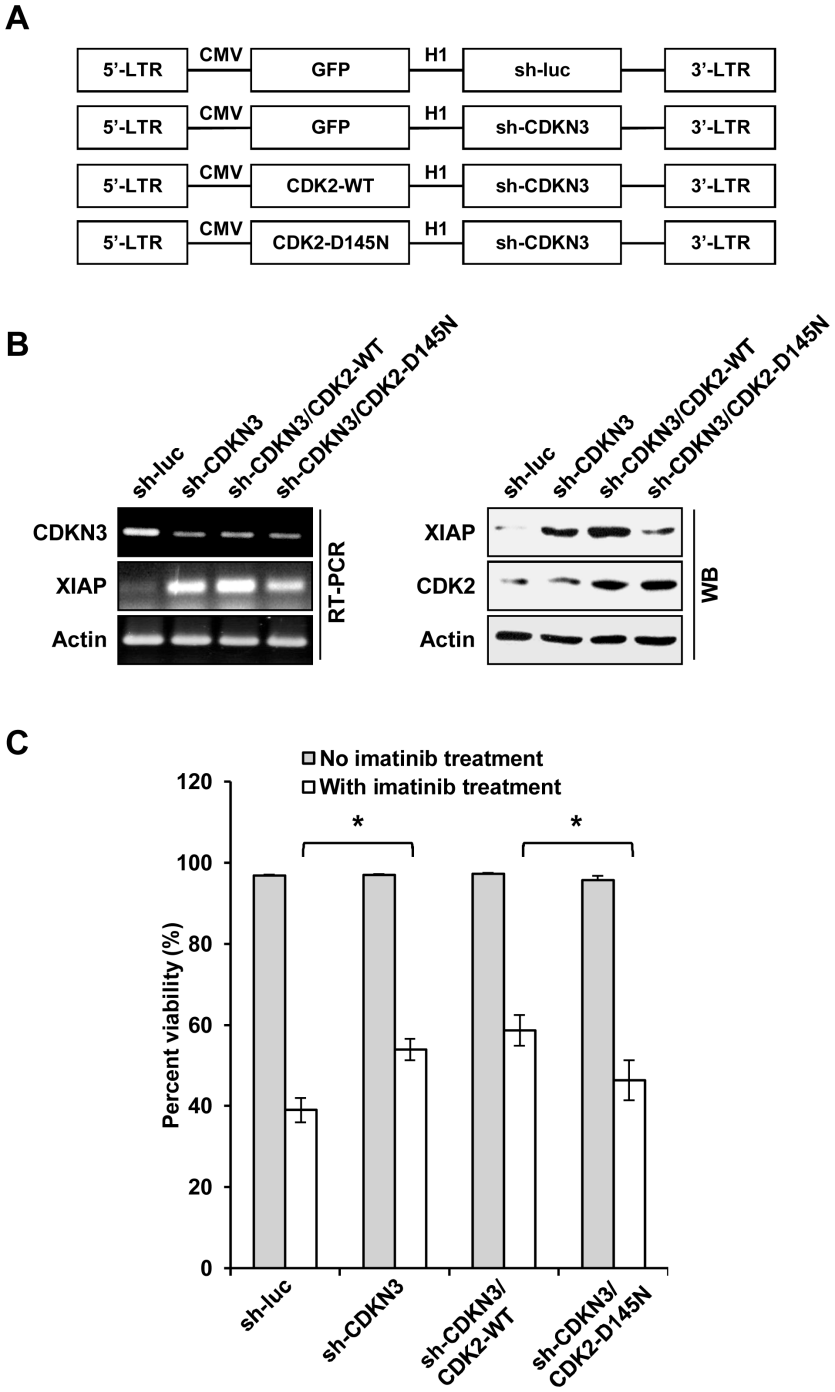
CDK2 is involved in regulating CDKN3-mediated leukemic cell survival. (A) Shown is lentiviral vectors constructed in this study that encode luciferase shRNA (sh-luc) control, CDKN3 shRNA (sh-CDKN3), sh-CDKN3 and either wild type CDK2 (CDK2-WT) or CDK2 dominant-negative mutant (CDK2-D145N). (B) RT-PCR and Western blotting were performed to examine the expression of CDKN3, CDK2, and XIAP in K562 cells expressing sh-CDKN3 alone, sh-CDKN3 and CDK2-WT, sh-CDKN3 and CDK2-D145N, or control. (C) Survival of K562 cells expressing sh-CDKN3, sh-CDKN3 and CDK2-WT, sh-CDKN3 and CDK2-D145N, or the control was analyzed by flow cytometry after treatment with or without 10 µM of imatinib for 48 h. Plotted are results from three independent experiments. Error bars represent SEM, *n* = 3; *P<0.05.

To confirm the above findings, we generated CDKN3-knockdown K562 cells stably expressing either CDK2-WT, CDK2-D145N (dominant-negative mutant), or the control (Figures 6A and 6B). These cells were subjected to imatinib treatment and analyzed for cell survival. As shown in Figure 6C, without imatinib treatment, depletion of CDKN3, overexpression of CDK2-WT or CDK2-D145N did not significantly affect cell viability in K562 cells as compared to the control cells. However, consistently with our aforementioned findings, depletion of CDKN3 significantly increased the survival of imatinib-treated leukemic cells (Figure 6C). Overexpression of functional CDK2 further increased the survival of CDKN3-depleted leukemic cells after treatment with imatinib, whereas ectopic expression of the catalytically inactive CDK2-D145N mutant had the opposite effect on survival of the CDKN3-deficient leukemic cells exposed to imatinib. Together, these results reveal that activity of CDK2 regulated by CDKN3 is involved in imatinib-induced apoptosis in K562 leukemic cells.

### Overexpression of CDKN3 delays the G1/S transition in K562 leukemic cells

**Figure 7:**
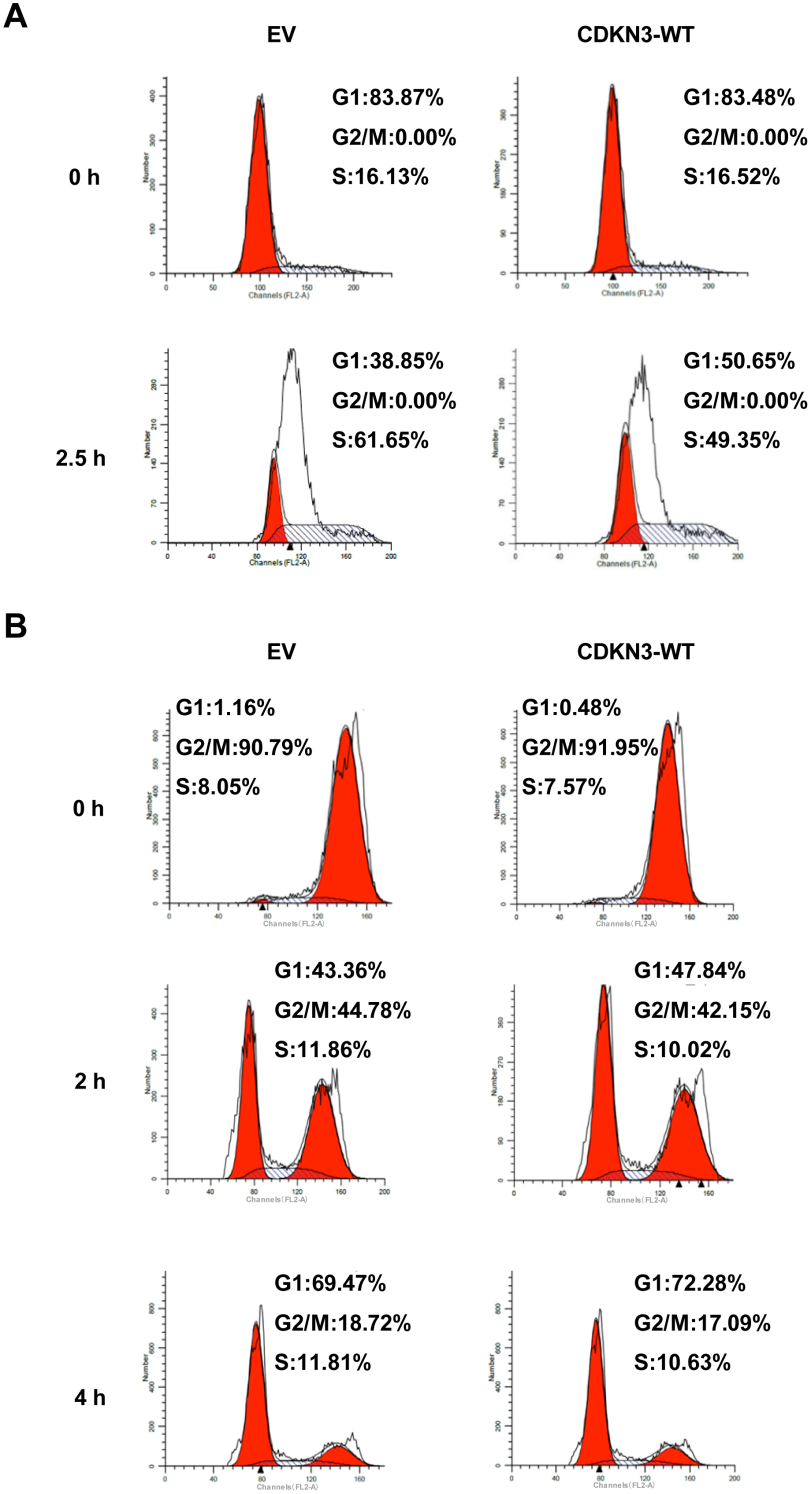
Overexpression of CDKN3 delays S phase entry in K562 leukemic cells. (A) K562 cells expressing CDKN3-WT or EV (control) were arrested at G1/S boundary by treatment with thymidine (2 mM) for 13.5 h and then released for 9 h followed by treatment with thymidine (2 mM) for 13.5 h. Cell cycle profiles at 0 and 2.5 h after release from thymidine treatment were analyzed by flow cytometry. (B) K562 cells expressing CDKN3-WT or EV control were arrested at G2/M phase by 2 mM of thymidine treatment for 13.5 h and then released for 6 h followed by treatment with 100 ng/ml of nocodazole for 6 h. Cell cycle profiles at 0, 2, and 4 h after release from nocodazole treatment were examined by flow cytometry.

Given that CDKN3 is a critical inhibitor for CDK2 [14], [19], we next evaluated the effect of CDKN3 on cell cycle progression in K562 cells. K562 cells stably expressing CDKN3-WT or control cells were synchronized at the G1/S boundary by thymidine treatment, and then subjected to cell cycle analysis (Figure S4A). As shown in Figure 7A, 61.65% of control cells resided in S phase, whereas only 49.35% of CDKN3-WT overexpressing cells were in S phase. This finding demonstrates that overexpression of CDKN3 delays the G1/S transition in K562 leukemic cells. Because CDKN3 also interacts with CDK1 [17], we further examined whether overexpression of CDKN3 affects the timing of mitotic exit upon nocodazole release in K562 cells (Figure S4B). As shown in Figure 7B, 18.7% of the control cells were labeled as G2/M phase population at 4 hours after release. Similarly, 17.1% of CDKN3 overexpressing cells were in G2/M phase at this time point, indicating that overexpression of CDKN3 had no significant effect on the timing of the G2/M/G1 progression upon release from nocodazole-induced mitotic spindle checkpoint arrest. Together, these data reveal that enforced expression of CDKN3 delays the G1/S transition but has little effect on G2/M/G1 progression in Bcr-Abl positive K562 cells.

## Discussion

Abnormal expression of CDKN3 is associated with a broad spectrum of human cancers. Dual roles of CDKN3 acting either as an oncogene or a tumor suppressor have been documented in different tumors [17], [20], [21], [23]. However, the functional relevance of CDKN3 in Bcr-Abl-mediated leukemias remains elusive. Here, for the first time, we present evidence that CDKN3 plays a crucial role in Bcr-Abl-mediated tumorigenesis. Overexpression of CDKN3 markedly sensitized K562 leukemic cells to imatinib-induced apoptosis, and inhibited Bcr-Abl-dependent tumor growth in nude mice. On the contrary, silencing CDKN3 greatly rendered K562 cells resistant to imatinib-induced apoptosis, and promoted K562 xenografted tumor growth in nude mice. In addition, overexpression of CDKN3 remarkably inhibited the transformation efficiency of FDCP1 cells induced by Bcr-Abl. These results strongly indicate that CDKN3 acts as a tumor suppressor in Bcr-Abl-mediated tumorigenesis.

Numerous studies have demonstrated that tumorgenesis induced by the oncogenic Bcr-Abl kinase is associated with dysregulation of a variety of signaling pathways, which endows leukemic cells with malignant proliferation and defected apoptosis [9], [37], [38]. It is well known that CDKN3 is a key inhibitor of CDK2 [15]. CDK2 controls multiple cell cycle checkpoints, including the G1/S transition and mitotic entry [39]–[41]. Activation of CDK2 requires the binding of cyclin E/A, but also requires phosphorylation of CDK2 at Thr160 by CDK-activating kinase (CAK) [39]–[41]. CDKN3 can form a stable complex with CDK2, dephosphorylating Thr160 and thus inactivating CDK2 [42]. Our experiments demonstrated that overexpression of CDKN3 was sufficient to prevent K562 leukemic cells from entering S phase of the cell cycle, suggesting that CDKN3 may negatively regulate proliferation of the leukemic cells by inactivating CDK2 and thereby delaying the S-phase entry. This finding is consistent with previously published observations in other experimental systems, implicating CDKN3 as a S-phase gatekeeper in multiple cell types [14]–[15], [19].

In this study, we also investigated the mechanisms by which CDKN3 promotes death of Bcr-Abl-driven leukemic cells upon imatinib exposure. Our data showed that overexpression of wild type CDKN3 significantly enhanced imatinib-induced apoptosis in K562 cells. We found that ectopic expression of the phosphatase-dead C140S CDKN3 mutant did not promote apoptosis under the same conditions. In addition, enforced expression of the CDK2 dominant-negative mutant (CDK2-D145N) attenuated survival of the CDKN3-knockdown leukemic cells, suggesting that increased CDK2 activity is essential for increased survival of the CDKN3-knockdown leukemic cells.

Several studies have revealed that XIAP, an anti-apoptotic protein regulated by CDK2, plays an important role in controlling cell survival [35], [36], [43], [44]. Our results demonstrated that the expression level of XIAP strongly correlated with the phosphorylation status of CDK2 and the expression of CDKN3 in the K562 leukemic cells, supporting the notion that CDKN3 may negatively regulate leukemic cell survival by dephosphorylating CDK2, leading to decreased expression of XIAP. Taken together, our observations suggested that CDKN3 suppresses Bcr-Abl-induced tumorigenesis likely through cell cycle arrest at the G1/S phases and regulation of the apoptosis by altering the XIAP expression.

In summary, our results reveal that CDKN3 acts as a tumor suppressor during Bcr-Abl-mediated tumorigenesis through control of both cell proliferation and cell survival. Downregulation or mutation of CDKN3 may increase cell proliferation and confer high resistance to imatinib-induced apoptosis in Bcr-Abl-positive leukemic cells. However, further studies are needed to address the precise mechanisms by which CDKN3 impacts these processes. In addition, future work will also explore the proposed role of inactivation of the CDKN3 tumor suppressor pathway in human leukemia. Whether this pathway has any prognostic or therapeutic significance in the leukemia induced by Abl oncogenes also remains to be determined.

## Supporting Information

Figure S1 **Generation of K562 cell lines stably expressing CDKN3, CDKN3 shRNA, or the controls.** (A) Generation of K562 cells stably overexpressing CDKN3-WT or empty vector (EV). Retroviruses encoding CDKN3-WT or EV were produced in 293T cells. Cell culture supernatants containing retroviruses were collected and filtered through a 0.22-µm MCE membrane (Millipore). K562 cells were infected with the retroviruses and GFP-positive K562 cells were sorted by flow cytometry. Shown are micrographs of these K562 cell lines obtained from a fluorescent microscope (Axiovert 200M; Zeiss, Oberkochen, Germany). (B) Experiments were performed as described in (A). Shown are micrographs of K562 cell lines stably expressing shRNA targeting CDKN3 or luciferase control obtained from a fluorescent microscope (Axiovert 200M; Zeiss, Oberkochen, Germany). (C) shRNA-based knockdown of CDKN3 was examined by real-time PCR in K562 cells expressing specific shRNAs. Plotted are results from three independent experiments. Error bars, SEM; *n* = 3; *P<0.05. (D) RT-PCR was performed to examine CDKN3 mRNA levels in cells described in (C).
(TIF)

Figure S2 **CDKN3 promotes K562 cell apoptosis induced by imatinib.** (A) K562 cells stably overexpressing CDKN3-WT or empty vector (EV) were stained with Annexin V-APC and PI, examined by flow cytometry and analyzed by FCS Express V3. Plotted are results from three independent experiments. Error bars represent SEM, *n* = 3. (B) K562 cells described in (A) were treated with 5 µM imatinib for 36 h. Samples were analyzed as described in (A). Plotted are results from three independent experiments. Error bars, SEM; *n* = 3; *P<0.05. (C) Experiments were performed as described in (A). Plotted are the results from three independent experiments using K562 cells expressing shRNA against CDKN3 or luciferase. Error bars, SEM; *n* = 3. (D) K562 cells expressing shRNA against CDKN3 or luciferase were treated with 5 µM imatinib for 36 h. Samples were analyzed as described in (A). Plotted are results from three independent experiments. Error bars represent SEM, *n* = 3; *P<0.05.
(TIF)

Figure S3 **Disruption of CDKN3 expression increased the survival of Bcr-Abl positive SUP-B15 cell.** (A) The mRNA expression of CDKN3 in K562 and SUP-B15 cells were measured by RT-PCR. (B) shRNA-based knockdown of CDKN3 was examined in SUP-B15 cells expressing shRNA targeting CDKN3 or luciferase by real-time PCR. Shown are results from three independent experiments. Error bars, SEM; *n* = 3; *P<0.05. (C) SUP-B15 cells described in (B) were treated with 5 µM imatinib for 24 h and stained with Annexin V-APC/PI. Samples were analyzed by flow cytometry and FCS Express V3. Plotted are results from three independent experiments. Error bars, SEM; *n* = 3; *P<0.05.
(TIF)

Figure S4 **Schematic view of experimental design to examine the impact of CDKN3 on cell cycle progression.** (A) K562 cells expressing CDKN3-WT or empty vector (EV) were treated with thymidine (2 mM) for 13.5 h, released for 9 h and then treated with thymidine (2 mM) for 13.5 h again. Cells were released for 2.5 h and subjected to flow cytometry analysis. (B) Cells described in (A) were treated by 2 mM of thymidine for 13.5 h, released for 6 h, and then treated by 100 ng/ml of nocodazole for 6 h. Then cells were released for indicated time and examined by flow cytometry.
(TIF)
