# Supplementary figures and images for: A Critical Role of CDKN3 in Bcr-Abl-Mediated Tumorigenesis
Source: PLoS One. 2014 Oct 31;9(10):e111611. doi: 10.1371/journal.pone.0111611 (PMC4216094; doi:10.1371/journal.pone.0111611)

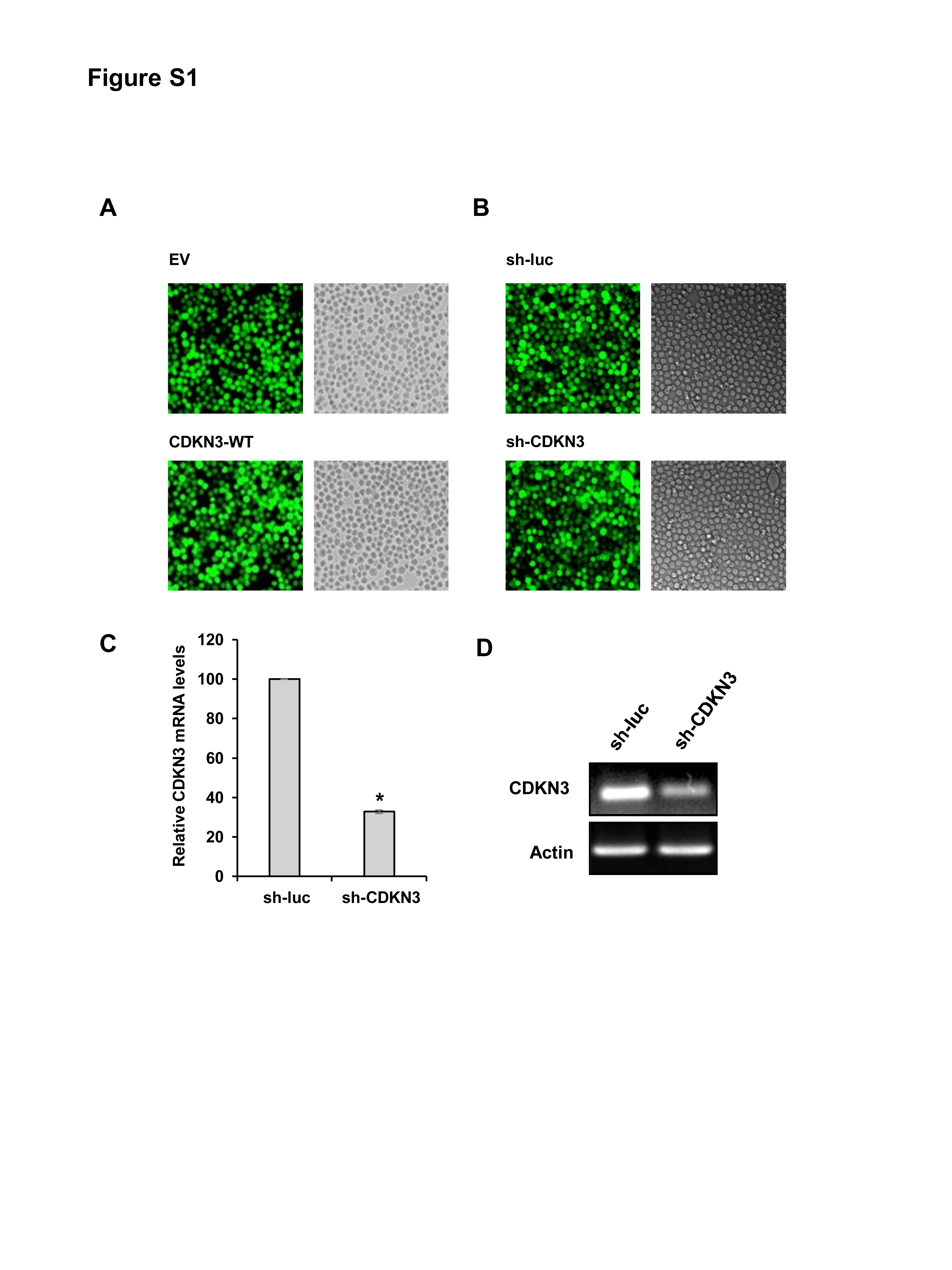

Supplement: Figure S1 — Generation of K562 cell lines stably expressing CDKN3, CDKN3 shRNA, or the controls. (A) Generation of K562 cells stably overexpressing CDKN3-WT or empty vector (EV). Retroviruses encoding CDKN3-WT or EV were produced in 293T cells. Cell culture supernatants containing retroviruses were collected and filtered through a 0.22-µm MCE membrane (Millipore). K562 cells were infected with the retroviruses and GFP-positive K562 cells were sorted by flow cytometry. Shown are micrographs of these K562 cell lines obtained from a fluorescent microscope (Axiovert 200M; Zeiss, Oberkochen, Germany). (B) Experiments were performed as described in (A). Shown are micrographs of K562 cell lines stably expressing shRNA targeting CDKN3 or luciferase control obtained from a fluorescent microscope (Axiovert 200M; Zeiss, Oberkochen, Germany). (C) shRNA-based knockdown of CDKN3 was examined by real-time PCR in K562 cells expressing specific shRNAs. Plotted are results from three independent experiments. Error bars, SEM; n = 3; *P<0.05. (D) RT-PCR was performed to examine CDKN3 mRNA levels in cells described in (C). (TIF) [file pone.0111611.s001.tif]

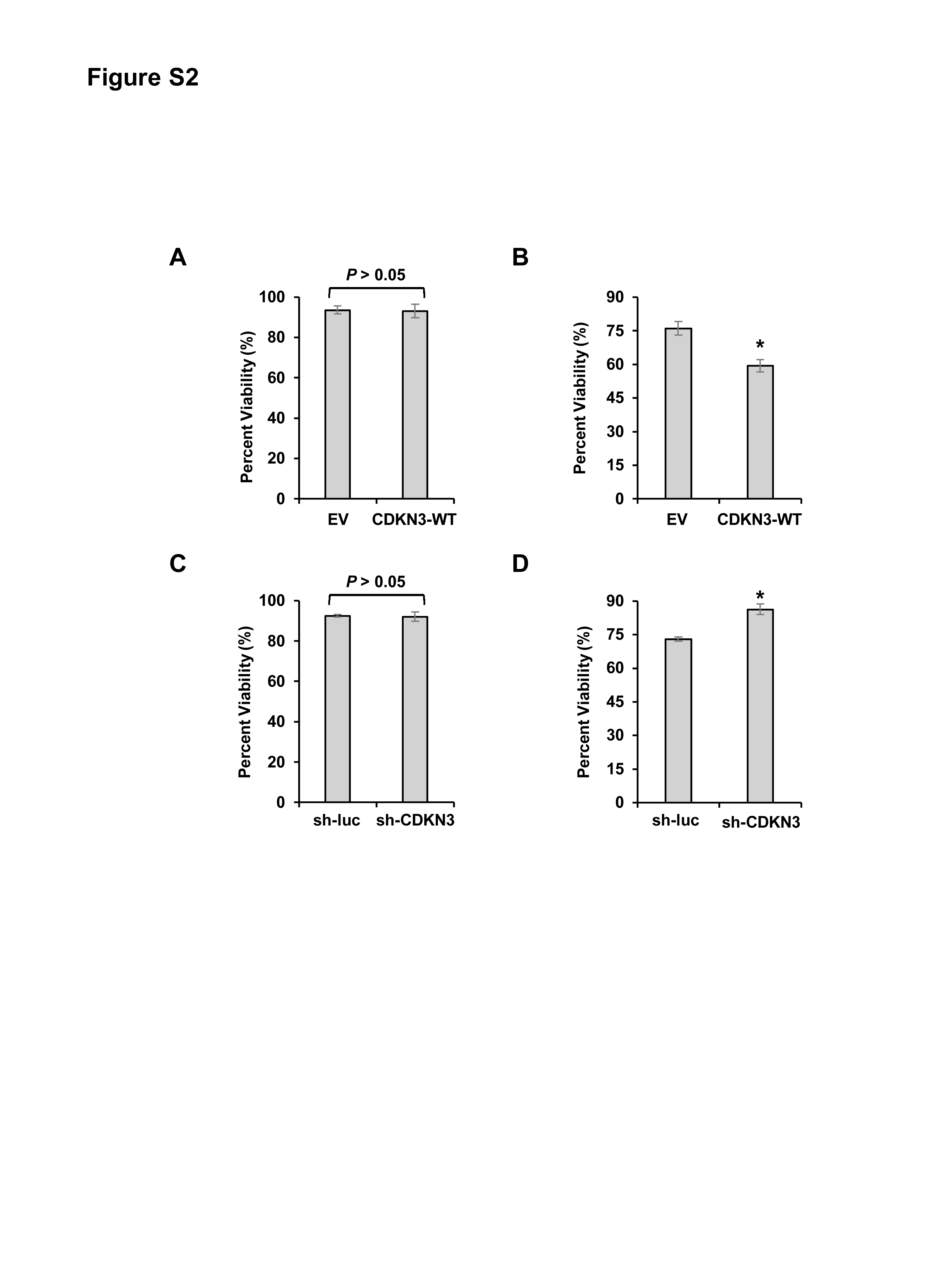

Supplement: Figure S2 — CDKN3 promotes K562 cell apoptosis induced by imatinib. (A) K562 cells stably overexpressing CDKN3-WT or empty vector (EV) were stained with Annexin V-APC and PI, examined by flow cytometry and analyzed by FCS Express V3. Plotted are results from three independent experiments. Error bars represent SEM, n = 3. (B) K562 cells described in (A) were treated with 5 µM imatinib for 36 h. Samples were analyzed as described in (A). Plotted are results from three independent experiments. Error bars, SEM; n = 3; *P<0.05. (C) Experiments were performed as described in (A). Plotted are the results from three independent experiments using K562 cells expressing shRNA against CDKN3 or luciferase. Error bars, SEM; n = 3. (D) K562 cells expressing shRNA against CDKN3 or luciferase were treated with 5 µM imatinib for 36 h. Samples were analyzed as described in (A). Plotted are results from three independent experiments. Error bars represent SEM, n = 3; *P<0.05. (TIF) [file pone.0111611.s002.tif]

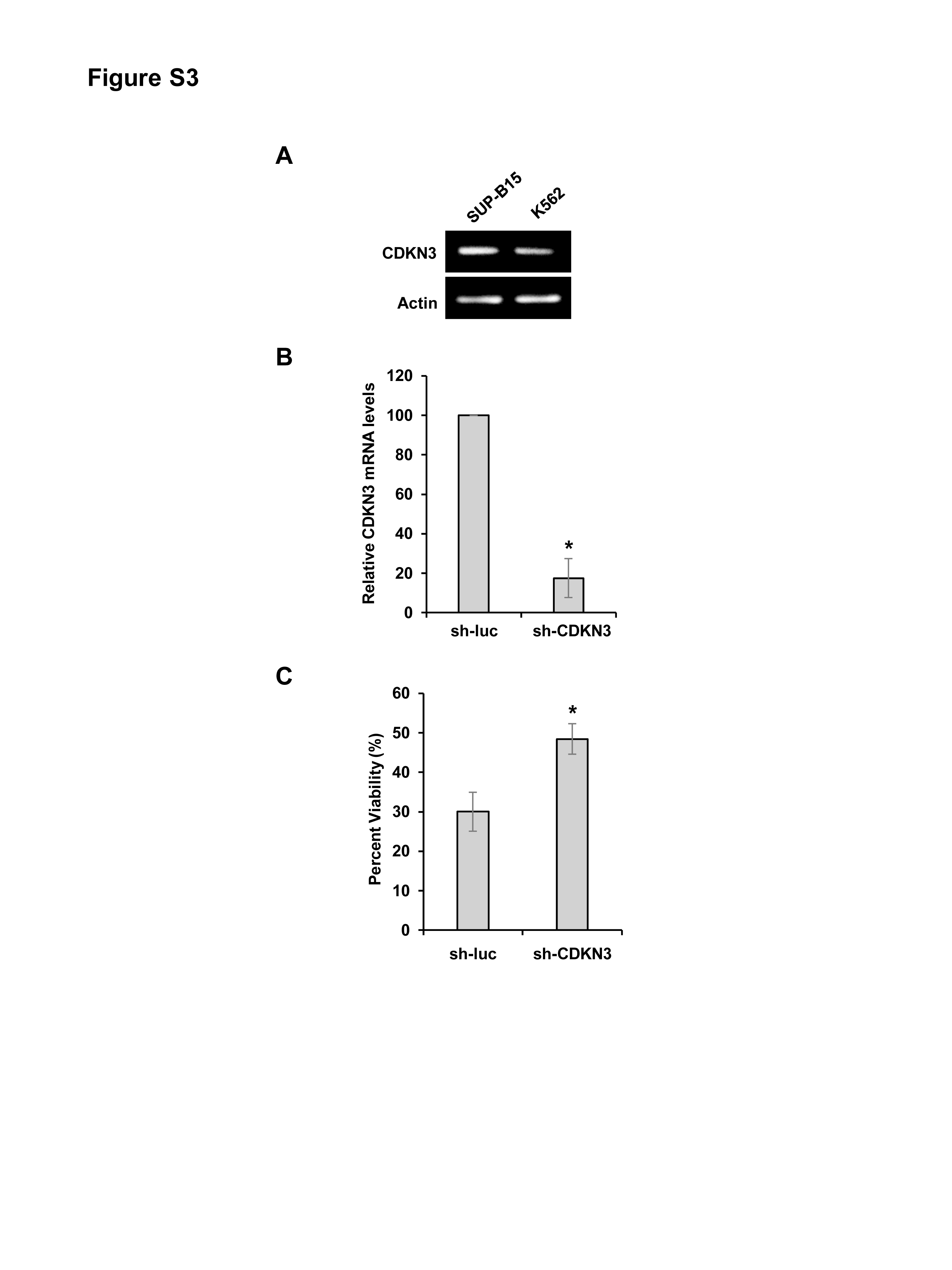

Supplement: Figure S3 — Disruption of CDKN3 expression increased the survival of Bcr-Abl positive SUP-B15 cell. (A) The mRNA expression of CDKN3 in K562 and SUP-B15 cells were measured by RT-PCR. (B) shRNA-based knockdown of CDKN3 was examined in SUP-B15 cells expressing shRNA targeting CDKN3 or luciferase by real-time PCR. Shown are results from three independent experiments. Error bars, SEM; n = 3; *P<0.05. (C) SUP-B15 cells described in (B) were treated with 5 µM imatinib for 24 h and stained with Annexin V-APC/PI. Samples were analyzed by flow cytometry and FCS Express V3. Plotted are results from three independent experiments. Error bars, SEM; n = 3; *P<0.05. (TIF) [file pone.0111611.s003.tif]

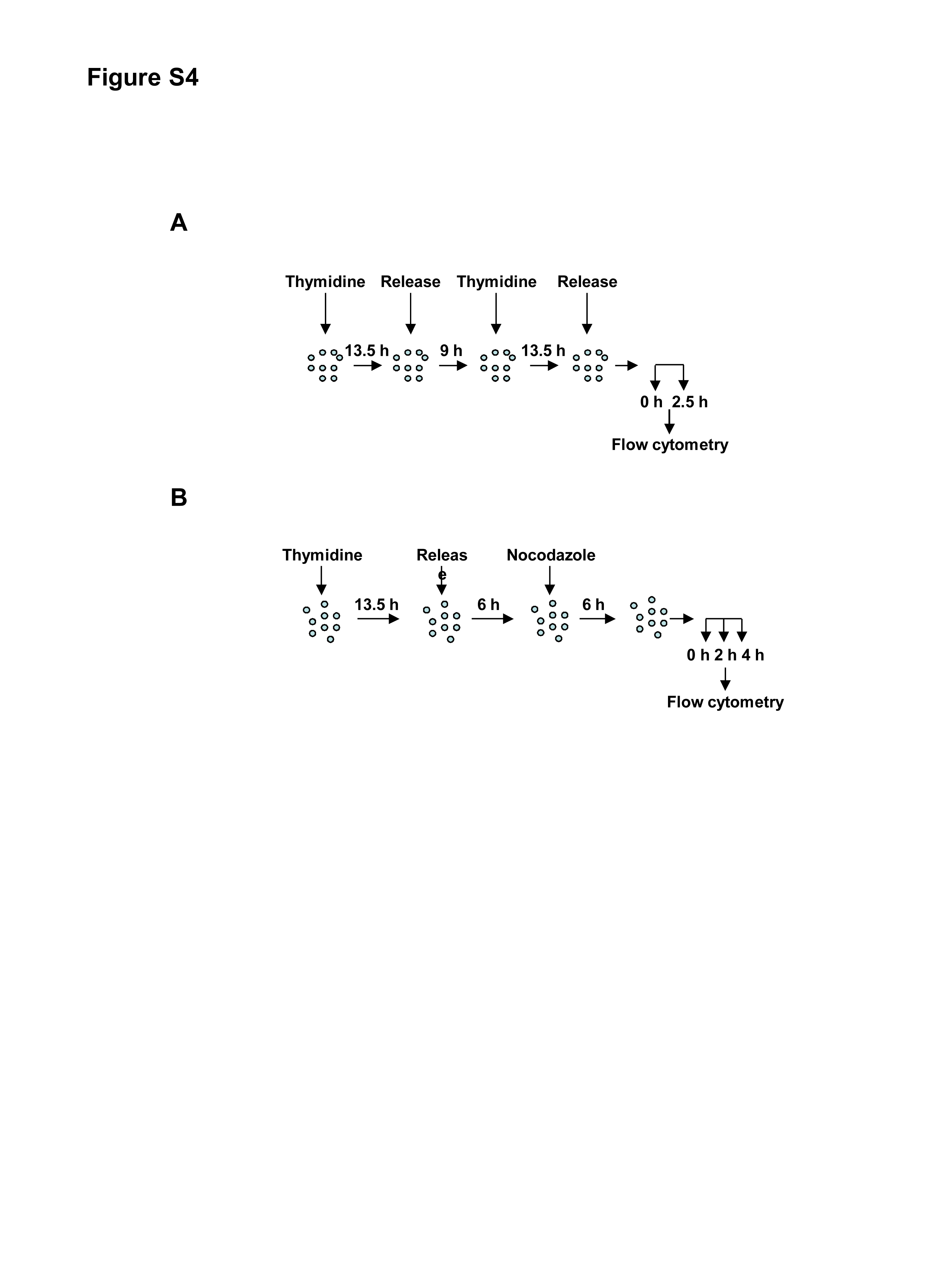

Supplement: Figure S4 — Schematic view of experimental design to examine the impact of CDKN3 on cell cycle progression. (A) K562 cells expressing CDKN3-WT or empty vector (EV) were treated with thymidine (2 mM) for 13.5 h, released for 9 h and then treated with thymidine (2 mM) for 13.5 h again. Cells were released for 2.5 h and subjected to flow cytometry analysis. (B) Cells described in (A) were treated by 2 mM of thymidine for 13.5 h, released for 6 h, and then treated by 100 ng/ml of nocodazole for 6 h. Then cells were released for indicated time and examined by flow cytometry. (TIF) [file pone.0111611.s004.tif]
